# Turkish adaptation, validation and reliability of the US Adult Food Security Survey Module in university students

**DOI:** 10.1017/S1368980024000223

**Published:** 2024-01-22

**Authors:** Yasemin Açar, Betül Sukan Karaçağıl, Meleknur Demirkoparan, Betül Şeref, Zeynep Kalaycı, Ayda Uçar, Hilal Yıldıran

**Affiliations:** 1 Department of Nutrition and Dietetics, University of Gazi, Ankara, Turkey; 2 Department of Nutrition and Dietetics, University of Karamanoglu Mehmetbey, Karaman, Turkey; 3 Department of Nutrition and Dietetics, University of Antalya Bilim, Antalya, Turkey

**Keywords:** Food security, Reliability, Validity, Survey, Adult Food Security Survey Module

## Abstract

**Objective:**

To evaluate the validity and reliability of the Turkish version of the US Adult Food Security Survey Module (AFSSM).

**Design:**

A cross-sectional study collected data from 117 university students. The AFSSM questionnaire was completed by all participants. Psychometric evaluation for scale, content, construct, and convergent validity and reliability of the scale was tested. The construct validity was assessed by exploratory factor analysis (EFA) and confirmatory factor analysis (CFA) on data collected from university students. Cronbach’s *α* (internal consistency) and composite reliability were used to assess the reliability (*P* < 0·05).

**Setting:**

Students were recruited from the university.

**Participants:**

This research was conducted with volunteer university students with a mean age of 22·74 ± 4·19 years.

**Results:**

Three factors were extracted from eight items through EFA: (1) inadequate nutrition, (2) economic concern and (3) hunger. These factors accounted for 77·4 % of the total variance, and factor loadings ranged from 0·755 to 0·953. Cronbach’s *α* was 0·769. The results of the CFA suggested the fit indices were acceptable (χ^2^/s d = 0·235, root mean error of approximation: 0·034, goodness-of-fit index: 0·994, comparative fit index: 0·992 and normed fit index: 0·986).

**Conclusions:**

This is the first study that validates and reports the Turkish version of AFSSM in university students, and the results of our study show that the Turkish AFSSM is a valid and reliable tool for determining food security in university students. AFSSM can be used by researchers to examine the food security of university students.

Food security is defined as ‘when all people at all times have physical, social and economic access to food, which is safe and consumed in sufficient quantity and quality to meet their dietary needs and food preferences, and is supported by an environment of adequate sanitation, health services, and care, allowing for a healthy and active life’ by the FAO in 2012^(1)^.

Food security is a multidimensional phenomenon and is covered under four dimensions. These are availability, access, utilisation and stability. Availability is related to the supply of food and means the availability of sufficient quality and variety. Access covers the access to these foods in economic and physical terms. Utilisation is how the body uses nutrients and includes topics such as food preparation or cooking. Stability, however, briefly expresses the continuity of these three dimensions^(2)^.

According to the State of World Food Security and Nutrition report published in 2021, undernourishment increased from 8·4 % to 9·9 % from 2019 to 2020, which means that in 2020 – 161 million more people than in 2019 – an average of 763 million people in the world will face hunger. One in three people (2·37 billion) worldwide did not have access to adequate food in 2020^(3)^. Ending hunger, achieving food security, improving nutrition and promoting sustainable agriculture are also part of sustainable development goals. Therefore, measuring and monitoring food security in valid and reliable ways is critical in tracking progress towards the Sustainable Development Goal (SDG) 2 for zero hunger^(4,5)^.

Many methods exist to measure food insecurity^(6,7)^, including experience-based food insecurity measurement tools^(8,9)^. Experience-based food insecurity scales emerged from the problems in the analysis of food consumption data and the need to address the concept of food insecurity more broadly. Experience-based food insecurity scales offer advantages in that they offer the possibility to directly capture the food insecurity situation and analyse it from a behavioural perspective^(10)^.

The eighteen-item Household Food Security Survey Module (HFSSM), developed in the USA^(7)^, later began to be implemented in surveys conducted for the US Department of Agriculture (USDA) in 1995^(11)^. Apart from HFFSM, there are shorter scales developed specifically for adults and children. The Adult Food Security Survey Module (AFSSM), which consists of ten items, does not provide specific information on children’s food security, but the screening keeps the respondent burden to the minimum needed to get reliable data^(12)^. The US Food Security Survey Modules have been adapted for use in a wide variety of cultural and linguistic settings worldwide^(13–16)^. When we searched the literature, it was seen that there was no Turkish validity and reliability study of the AFSSM. In addition, we did not find a similar scale that measures food security at the individual level in Turkey. Therefore, due to the importance of the subject, this study was conducted to adapt the AFFSM, a food security scale, to the Turkish language culture and to test its validity and reliability.

## Materials and methods

### Aim

This study aimed to adapt the AFFSM to the Turkish language culture and evaluate its psychometric properties.

### Study design

This study established the AFSSM through a four-step process: (1) testing the validity (content, construct and language) of the scale (2), testing the reliability of the scale (3), exploratory factor analysis (EFA) and (4) confirmatory factor analysis (CFA). Data for the study were collected between May 2022 and June 2022.

**Fig. 1:**
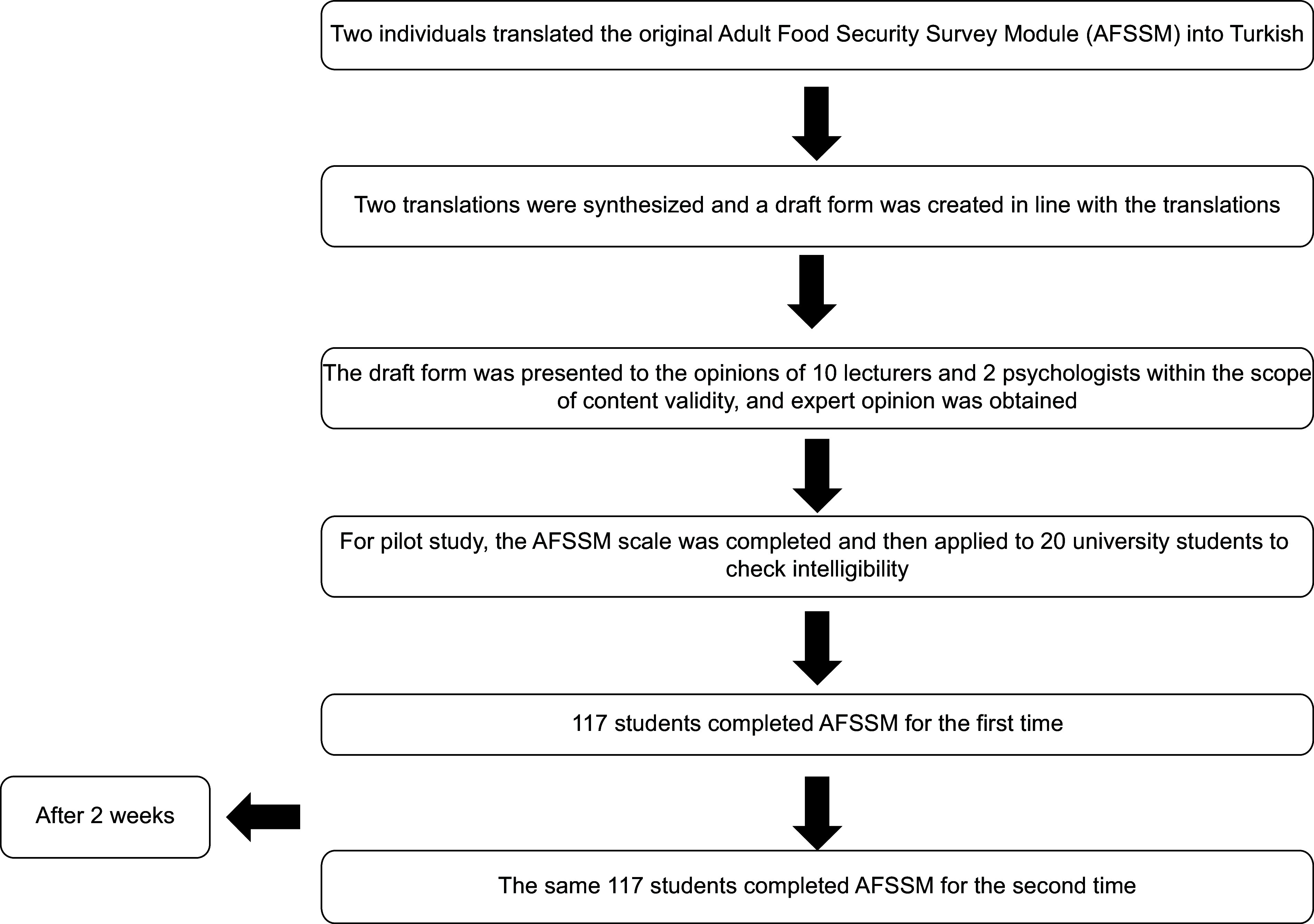
The flow chart of the adaptation stage

The AFSSM was translated from English to Turkish by two experts with proficiency in English and then from Turkish to English by another expert to ensure language validity. The draft form was created by considering the suggestions in the translations. For the content validity of the draft form, the expert opinions of ten lecturers in the Nutrition and Dietetics department and two psychologists were consulted. Davis technique was used for content validity^(17)^. After expert opinions, the scale was revised based on their comments, and the Turkish version of the scale was created by testing. The flow chart of the adaptation stages is shown in Fig. 1.

### Participants

The population of the study consists of university students. Study participants were enrolled using the following inclusion criteria: (a) being 18 years and older, (b) being enrolled as a student at a university, (c) using the Turkish language for communication and (d) volunteering to participate in the study. The exclusion criteria were as follows: (a) international students, (b) those under the age of 18 years and (c) non-university students.

Before the study, the participants were informed about the study, and their consent was obtained. According to the literature, the sample should be at least five or ten times the number of scale items to make factor analysis in validity and reliability studies^(18,19)^. Therefore, about 100 university students attained by calculating ten times the number of items (ten items) on the scale. For the pilot study, the AFSSM questionnaire was completed and then applied to twenty university students to check intelligibility. For the test–retest, 117 participants completed the AFSSM again 2 weeks after the first administration.

### Adult Food Security Survey Module

The Adult Food Security Survey Module consists of ten questions in total. Food security status of each household has been researched in the last 12 months. It is assessed through responses to ten closed-ended questions about food-related behaviours, experiences and conditions, which are known to characterise households that have difficulty meeting their food needs. The total score varies between 0 and 10 and is obtained from the sum of the number of affirmative answers given to the scale questions. Food insecurity is used to refer to both low and very low food security categories^(12)^. The items of the scale are presented in Table 1.

**Table 1:** The items of the AFSSM

Item	Questions
1	*‘(I/We) worried whether (my/our) food would run out before (I/we) got money to buy more’. Was that often true, sometimes true, or never true for (you/your household) in the last 12 months?*
	Often true
	Sometimes true
	Never true
	DK or refused
2	*‘The food that (I/we) bought just didn’t last, and (I/we) didn’t have money to get more’. Was that often, sometimes, or never true for (you/your household) in the last 12 months?*
	Often true
	Sometimes true
	Never true
	DK or refused
3	*‘(I/we) couldn’t afford to eat balanced meals’. Was that often, sometimes, or never true for (you/your household) in the last 12 months?*
	Often true
	Sometimes true
	Never true
	DK or refused
4	*In the last 12 months, since last (name of current month), did (you/you or other adults in your household) ever cut the size of your meals or skip meals because there wasn’t enough money for food?*
	Yes
	No (Skip 5)
	DK (Skip 5)
5	*(If yes above, ask) How often did this happen – almost every month, some months but not every month, or in only 1 or 2 months?*
	Almost every month
	Some months but not every month
	Only 1 or 2 months
	DK
6	*In the last 12 months, did you ever eat less than you felt you should because there wasn’t enough money for food?*
	Yes
	No
	DK
7	*In the last 12 months, were you every hungry but didn’t eat because there wasn’t enough money for food?*
	Yes
	No
	DK
8	*In the last 12 months, did you lose weight because there wasn’t enough money for food?*
	Yes
	No
	DK
9	*In the last 12 months, did (you/you or other adults in your household) ever not eat for a whole day because there wasn’t enough money for food?*
	Yes
	No (Skip 10)
	DK (Skip 10)
10	*(If yes above, ask) How often did this happen – almost every month, some months but not every month, or in only 1 or 2 months?*
	Almost every month
	Some months but not every month
	Only 1 or 2 months
	DK

AFSSM, Adult Food Security Survey Module; DK, don’t know.

### Ethical considerations

The Ethics Commission at Gazi University approved this study (code: 2022-625/10-05-2022). Before starting the study, an informative text about the study was presented to the participants and the consent of the participants was obtained. The study was conducted by the principles of the Declaration of Helsinki.

### Data analysis

In the study, the validity and reliability of the Turkish version of AFSSM were tested. After checking the translation of the scale from English to Turkish, factor analysis was performed for its validity. The suitability of the dataset to the normal distribution was examined using the skewness and kurtosis values, and it was seen that the dataset was suitable for the normal distribution. The Bartlett test was used to decide the suitability of the data for factor analysis and the Kaiser–Meyer–Olkin test was used to test the suitability of the sample size for the factor analysis. EFA was used to analyse the construct validity of the scale. The dataset suitable for factor analysis was analysed using the principal component analysis method and the varimax rotation, which is one of the vertical rotation techniques, assuming that the factors are unrelated to each other based on the theoretical background in line with the recommendations of the literature^(19–21)^. Then, the number of factors of the tested structure and which items load which factors were analysed. CFA was used to analyse the compatibility of the subdimensions with the original scale and test the confirmability of the construct that emerged as a result of the EFA. The scale structure was examined in line with the fit indices suggested by the literature and the criterion values of these indices. The following indices were used to determine the model fit: *χ* ^2^ test, comparative fit index (CFI), goodness-of-fit index (GFI), normed fit index (NFI), adjusted goodness-of-fit Index (AGFI), root mean square error of approximation (RMSEA) and standardised root mean square residual (SRMR). The validity analysis was performed with the R program.

To determine the reliability of the scale, item analysis was performed for internal consistency, the reliability coefficient (Cronbach *α*) was calculated and the item-total test correlations of the items were examined. Factor scores were interpreted with analysed Cronbach’s *α* values. The reliability coefficient is between 0 and +1, and reliability was considered to be acceptable for 0·7 ≤ Cronbach’s *α* < 0·8, good for 0·8 ≤ Cronbach’s *α* < 0·9 and excellent for Cronbach’s *α* ≥ 0·9. Values close to 1 indicate that the reliability and internal consistency between the items are high^(22–24)^.

Mean and standard deviation values were given for descriptive statistics related to the demographic characteristics. SPSS 24.0 program was used in the evaluation of the data, and the level of significance was accepted as *P* < 0·05. The test–retest reliability of the scale was re-evaluated 2 weeks later.

## Results

In this study, 117 university students (10 males and 107 females) participated in the construct validity of AFSSM. Students studying at Gazi University, Department of Nutrition and Dietetics, were included in the study. The baseline characteristics of the participants are presented in Table 2. The mean age of students was 22·7 ± 4·1 years.

**Table 2:** Demographic characteristics of students

Characteristic	Group	*n*	%
Age			
Mean		22·74	
sd		4·19	
BMI category (kg/m^2^)	Underweight (<18·5)	10	8·5
	Normal (18·5–24·99)	84	71·8
	Pre-obesity (25·0–29·99)	23	19·7
BMI (kg/m^2^)			
Mean		22·15	
sd		3·58	
Family settlement	Rural	34	29·1
	Urban	83	70·9
Parent working status	Mother is working, the father is not working	2	1·7
	Father is working, the mother is not working	67	57·3
	Both are not working	26	22·2
	Both are working	16	13·7
	One of the parents is retired	6	5·1
Working status	Working	5	4·3
	Not working	112	95·7
Individual income status (₺)			
Mean		1711·97	
sd		1815·30	
Food expenditure status (₺)			
Mean		777·86	
sd		880·57	
Food expenditures / income ratio (₺)			
Mean		53·25	
sd		61·33	

₺: Turkish Lira.

Participants of 71·8 % had average body weight, and their mean BMI was 22·15 ± 3·58 kg/m^2^. Participants of 70·9 % were residing in the city, 57·3 % of their fathers working and their mothers not working. Participants of 4·3 % are working and, the income means were 1711·97 ± 1815·30 Turkish Lira (₺). Their food expenditures were 777·86 ± 880·57 ₺, and food expenditures/income ratio means were found 53·2 %.

### Validity

#### Content validity

For a content validity evaluation, a lecturer in the Nutrition and Dietetics department experienced scale development, and two psychologists were asked to comment on the grammar, wording and scoring of the AFSSM items with the Davis technique^(17)^. The scale was revised based on their comments.

#### Explanatory factor analysis

Bartlett’s test value was 450·653 (*P* = <0·001), and Kaiser–Mayer–Olkin test value was 0·686. Two items (I) (I7, I8) that the item load value is <0·50 and have a low factor load value were excluded from the scale. After two questions were removed from the scale, the process was repeated on the remaining eight questions. As a result of EFA, three factors were obtained with factor loadings below 0·50 and eigenvalues above 1. The eigenvalues of these three factors were 3·99, 1·54 and 1·25, respectively, and they explained 77·4 % of the total variance of the AFSSM (Table 3).

**Table 3:** Explanatory variance ratio and eigenvalue results of Turkish AFSSM

Component	Initial eigenvalues			Rotation sums of squared loadings		
	Total	% of variance	Cumulative %	Total	% of variance	Cumulative %
1	3·999	42·482	42·482	2·292	28·646	28·646
2	1·542	19·272	61·755	2·040	25·505	54·151
3	1·256	15·697	77·452	1·864	23·301	77·452
4	0·586	7·326	84·778			
5	0·513	6·411	91·189			
6	0·388	4·845	96·034			
7	0·195	2·453	98·487			
8	0·121	1·513	100·000			

AFSSM, Adult Food Security Survey Module.

The resulting factors were named ‘Factor-1: Inadequate Nutrition’, ‘Factor-2: Economic Concern’ and ‘Factor-3: Hunger’. The factor loadings, eigenvalues and explained percentages of variance of the items constituting these factors are given in Table 4. It is seen that the ‘Factor-1’ dimension consists of ‘I4, I5, I6’, ‘Factor-2’ dimension consists of ‘I1, I2, I3’ and ‘Factor-3’ dimension consists of ‘I9, I10’ items. The factor load, which shows the relationship of each item with the total score, was over 0·70, and three factors accounted for 65 % of the variance.

**Table 4:** Factor loading of the Turkish AFSSM after varimax rotation with three factors

Factor names		Component		
	Item	F1	F2	F3
Factor 1 inadequate nutrition	4	0·908		
	5	0·883		
	6	0·755		
Factor 2 economic concern	2		0·860	
	1		0·764	
	3		0·759	
Factor 3 Hunger	10			0·953
	9			0·937
Eigenvalues		2·554	2·109	1·855
Explained variance (%)		25·542	21·094	18·553

AFSSM, Adult Food Security Survey Module; F, factor; I, item.

Based on the factor levels obtained as a result of the EFA, the AFSSM has a mixed 3, 4, and 5 Likert types, eight-item, and three-factor structure.

#### Confirmatory factor analysis

In this study, before starting the CFA, it was checked whether the data showed normal distribution and whether there were any empty cells or missing data in the database. The covariance matrix was produced, and analyses were made since the data followed the normal distribution. The process of generating the matrix was done by the maximum likelihood estimation method by the R program. Then, the fit indexes of the model were evaluated^(25)^.

In the model, it was observed that the criterion values were met and the model created is given in Fig. 1. In addition, since there was no item with standardised factor loadings below 0·5, no items were removed from the scale. The fit of the final model obtained after this process was tested with fit indices such as *χ* ^2^/s d, AGFI, CFI, GFI, NFI, RMSEA and SRMR^(26)^. The results of the CFA and GFI indices are shown in Table 5.

**Table 5:** Confirmatory factor analysis fit index results of the Turkish version of the Turkish AFSSM

Fit statistics	Value	Criteria
*χ* ^2^/sd	0·235	0 ≤ *χ* ^2^/s d ≤ 2
AGFI (adjusted goodness of fit index)	0·986	0·90 < AGFI
CFI (comparative fit index)	0·992	0·90 < CFI
GFI (goodness-of-fit index)	0·994	0·90 < GFI
NFI (normed fit index)	0·986	0·90 < NFI
RMSEA (root mean squared error of approximation)	0·034	RMSEA < 0·08
SRMR (standardised root mean square residual)	0·041	SRMR < 0·08

AFSSM, Adult Food Security Survey Module.

As a result of the analysis, the GFI indices were as follows: *χ* ^2^/s d = 0·235, AGFI = 0·986, CFI = 0·992, GFI = 0·994, NFI = 0·986, RMSEA = 0·034 and SRMR = 0·041.

**Fig. 2:**
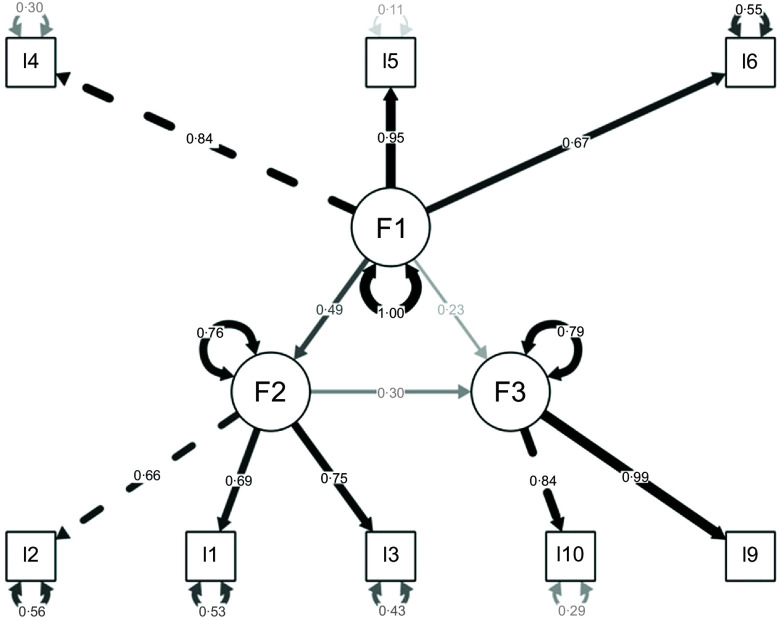
The CFA model of AFSSM

Based on the data obtained, it was concluded that the model fit values were at an acceptable level, and since most of the fit indices showed a high degree of fit, no change was made in the model structure. The CFA model of AFSSM is presented in Fig. 2.

#### Convergent validity

Convergent validity was used for the construct validity of the scale. Convergent validity means that statements about variables are related to each other and to the factors they generate. The composite reliability coefficient (composite reliability-CR) is used to evaluate convergent validity. The average variance extracted (AVE) value is the convergent validity between the items that make up a factor. For convergent validity, all CR values for the scale are expected to be greater than the AVE values and the AVE value to be greater than 0·5. In addition, the standardised factor loads of the items should be above 0·5 and the CR value should be higher than 0·7. The AVE value is obtained by dividing the sum of the squares of the item loads of the factor by the number of items^(27,28)^.

As a result of CFA, when convergent validity is examined (Table 6), it is seen that the standardised factor loads of the items are between 0·75 and 0·95; the CR and Cronbach’s *α* values are higher than 0·7 and the AVE values are higher than 0·5. Therefore, all three constructs have convergent validity. These results indicate that the scale used in the research has convergent validity.

**Table 6:** Component validity and reliability analysis results of Turkish AFSSM subfactors

Factors items		SFL (*λ*)*> 0·5	CR† > 0·7	AVE‡ > 0·5	Cronbach’s *α* > 0·7
Factor-1 Inadequate nutrition			0·89	0·73	0·761
	I4	0·908			
	I5	0·883			
	I6	0·755			
Factor-2 Economic concern			0·84	0·63	0·742
	I2	0·860			
	I1	0·764			
	I3	0·759			
Factor-3 Hunger			0·80	0·66	0·750
	I10	0·953			
	I9	0·937			

SFL, standardised factor loading; CR, composite reliability; AVE, average variance extracted; I, item.

* SFL (*λ*).

† CR.

‡ AVE.

### Reliability

#### Cronbach’s α coefficient – internal consistency

Internal consistency is based on the assumption that items measuring the same construct should be related to each other, and the most commonly used statistic to evaluate internal consistency is the Cronbach’s *α* coefficient. Cronbach’s *α* coefficients are between 0 and 1. A coefficient close to 1 means the scale is reliable and reflects better internal consistency^(29,30)^.

In this study, Cronbach’s *α* coefficient was determined as a result of the items (eight items) obtained after the EFA was found to be 0·769, supporting the scale reliability. Cronbach’s *α* values for ‘inadequate nutrition’, ‘economic concern’ and ‘hunger’ were found to be 0·761, 0·742 and 0·750, respectively, and ranged from 0·72 to 0·77 for the three dimensions (Table 7).

**Table 7:** Item-total correlation of Turkish AFSSM and Cronbach’s *α* coefficients when the item is deleted

Items	Scale mean if item deleted	Item-total correlation	Cronbach’s *α* if item deleted
1	23·44	0·51	0·73
2	23·30	0·50	0·74
3	23·61	0·52	0·73
4	24·14	0·66	0·72
5	21·81	0·62	0·74
6	24·16	0·56	0·73
7	23·99	0·35	0·76
8	23·92	0·14	0·77
9	23·98	0·44	0·76
10	21·07	0·29	0·76

AFSSM, Adult Food Security Survey Module.

#### Test–Retest

When the scale is applied to the same individuals at different times, getting similar answers indicates the stability of the scale. In this context, test–retest reliability is the most common method used to evaluate the stability of a scale^(31)^. To calculate the test–retest reliability of the scale, the questionnaire was applied again 2 weeks after the first application for the retest application. When the test–retest analysis correlations of the AFSSM dimension and total scores were examined, it was found that all dimensions and total scores of the scale showed a statistically significant positive correlation (*P* < 0·01; *P* < 0·001). When the test–retest results were examined, it was concluded that the scale was reliable and test–retest reliability was acceptable. The correlation coefficients are given in Table 8.

**Table 8:** Correlations between the three dimensions and the total score of Turkish AFSSM

Factors		F1 final test	F2 final test	F3 final test	F total final test
F1: pre-test	*r*	0·537	0·431	0·471	0·590
	*P*	<0·001**	<0·001**	0·004*	<0·001**
F2: pre-test	*r*	0·571	0·596	0·401	0·562
	*P*	<0·001**	<0·001**	0·033*	<0·001**
F3: pre-test	*r*	0·588	0·581	0·469	0·487
	*P*	<0·001**	<0·001**	0·004*	0·002*
F total: pre-test	*r*	0·524	0·588	0·419	0·669
	*P*	<0·001**	<0·001**	0·001*	<0·001**

AFSSM, Adult Food Security Survey Module; F, factor; *r*, Pearson’s correlation coefficients.

* *P* < 0.01.

** *P* < 0.001.

#### Scoring

The eight items of the AFSSM were scored on a 3, 4 and 5-Likert scale. The highest score of each question is 6, and the highest score of the scale is 48. The total score of the scale is between 0 and 48. The cut-off point of the scale is 10. The cut-off point of the scale was determined as a result of the Receiver Operating Characteristics (ROC) curve analysis. (0–10 = have food security and 11–48 = have no food security). The ROC curve analysis is presented in Table 9.

**Table 9:** The results of the ROC analysis

Variable	Cut-off	SEN	SPC	AUC	*P*
Income status	>10	0·368	0·352	0·625	0·011*
Food expenditure	>8·5	0·484	0·458	0·485	0·106

SEN, sensitive; SPC, specificity.

* *P* < 0.05.

## Discussion

In the study, the validity and reliability of the adaptation of AFFSM to the Turkish language and culture were tested. A total of 117 individuals were included in this study.

The AFFSM^(19)^ is a food security measurement tool used around the world by adapting it to various languages. The results of the scale adaptation studies have shown that the scale has good internal validity, and its use in different regions is reliable^(13–16)^.

The validity and reliability study of the short version of the Household Food Security Survey Module (HFFSM-SF)^(12)^ for Turkey was conducted by Emiral et al. in 2017^(32)^. However, based on our literature research, we found no scale measuring food security at the individual level in Turkey. Therefore, we tested the validity and reliability of the AFSSM by adapting it to the Turkish language and culture.

The scale includes differences according to the US methodology. According to the EFA, it was seen that the scale had a three-factor structure. One of the factors focuses on the relationship between food insecurity and inadequate nutrition, and the other focuses on its relationship with hunger. This distinction can help us learn more about individuals’ coping strategies for food insecurity. The other factor highlights the impact of economic concern and poverty on food access.

The original scale followed a categorisation method (very low food security, low food security and marginal food security) according to the presence and severity of food insecurity by scoring only on affirmative options. The US version calculates food insecurity based on yes questions only; this new scale improves sensitivity within frequencies and gives different scores to ‘yes’, ‘often’, ‘sometimes’, ‘almost every month’ and ‘some months but not every month’ answers. However, in some reports, it is reported that they are divided into two separate categories and defined as food-secure and food-insecure^(12)^. As in the results of the study of Wehler et al.^(33)^, the first study in which categories were discussed after Radimer et al.^(34)^, we also similarly found that the answers were not compatible for more than one category according to income status. Therefore, we propose, presenting the results in two categories, food-secure and food-insecure.

We are aware that the answers do not express results that are equidistant from each other. However, since we think that not every affirmative answer is equally important, we have adopted a scoring system based on the predominance of points for questions that have frequency options (Q1, Q2, Q3, Q5 and Q10). Our scale score range (0–48) thus differed compared with the original scale’s score range (0–10). However, thanks to this situation, we hope that more precise and sensitive results can be obtained in studies to be carried out, especially among samples with similar food security degrees.

Another difference between these two scales stems from the number of questions. Although the original scale consisted of ten questions, this adapted new scale consisted of eight questions. One of the questions with a low item load was ‘In the last 12 months, did you lose weight because there wasn’t enough money for food?’. We think that the reason why this problem has a low factor load is that the relationship between food security and body weight is still controversial^(35–38)^. Although the item load was not very low (0·491), the other question we asked was ‘In the last 12 months, were you ever hungry but didn’t eat because there wasn’t enough money for food’. We chose to exclude this question to obtain more consistent results due to the significant difference between the other item loads.

We could not perform a reference validity analysis because there is no other parallel scale measuring food security at the individual level in our country. However, this scale will allow future studies to perform this test. In addition, the population participating in the survey represents a limited population section regarding age and education level. Although we think there is ethnic diversity because university students are from various regions of the country, we recommend that the scale be re-evaluated for older and/or less educated people.

## Conclusion

The results of the study show that the AFFSM, which we have adapted to the Turkish language and culture, is valid and reliable. Thus, we brought the first Turkish Adult Food Security Scale, which measures food security individually, to the literature. We hope this scale will be a qualified tool to determine food insecurity in adults living in Turkey and reveal the impact of food insecurity on other parameters. Finally, we hope to support further studies to identify food security or conditions associated with food security.

## Limitations

Among the limitations of this study is that the participants included in the study are students of the Nutrition and Dietetics departments.
